# Silicone Composites with Electrically Oriented Boron Nitride Platelets and Carbon Microfibers for Thermal Management of Electronics

**DOI:** 10.3390/polym17020204

**Published:** 2025-01-15

**Authors:** Romeo Cristian Ciobanu, Magdalena Aflori, Cristina Mihaela Scheiner, Mihaela Aradoaei, Dorel Buncianu

**Affiliations:** 1 Department of Electrical Measurements and Materials, Gheorghe Asachi Technical University, 700050 Iasi, Romania; cschrein@tuiasi.ro (C.M.S.); mihaela.aradoaei@academic.tuiasi.ro (M.A.); 2 Petru Poni Institute of Macromolecular Chemistry, 41 A Gr. Ghica Voda Alley, 700487 Iasi, Romania; maflori@icmpp.ro; 3 Faculty of Mechanics, University Politehnica of Timisoara, Piata Victoriei 2, 300006 Timisoara, Romania; dorel.buncianu@upt.ro

**Keywords:** silicone composites, distributed boron nitride platelets, carbon microfibers, electrical characteristics, thermal conductivity, electronics thermal management

## Abstract

This study investigated silicone composites with distributed boron nitride platelets and carbon microfibers that are oriented electrically. The process involved homogenizing and dispersing nano/microparticles in the liquid polymer, aligning the particles with DC and AC electric fields, and curing the composite with IR radiation to trap particles within chains. This innovative concept utilized two fields to align particles, improving the even distribution of carbon microfibers among BN in the chains. Based on SEM images, the chains are uniformly distributed on the surface of the sample, fully formed and mature, but their architecture critically depends on composition. The physical and electrical characteristics of composites were extensively studied with regard to the composition and orientation of particles. The higher the concentration of BN platelets, the greater the enhancement of dielectric permittivity, but the effect decreases gradually after reaching a concentration of 15%. The impact of incorporating carbon microfibers into the dielectric permittivity of composites is clearly beneficial, especially when the BN content surpasses 12%. Thermal conductivity showed a significant improvement in all samples with aligned particles, regardless of their composition. For homogeneous materials, the thermal conductivity is significantly enhanced by the inclusion of carbon microfibers, particularly when the boron nitride content exceeds 12%. The biggest increase happened when carbon microfibers were added at a rate of 2%, while the BN content surpassed 15.5%. The thermal conductivity of composites is greatly improved by adding carbon microfibers when oriented particles are present, even at BN content over 12%. When the BN content surpasses 15.5%, the effect diminishes as the fibers within chains are only partly vertically oriented, with BN platelets prioritizing vertical alignment. The outcomes of this study showed improved results for composites with BN platelets and carbon microfibers compared to prior findings in the literature, all while utilizing a more straightforward approach for processing the polymer matrix and aligning particles. In contrast to current technologies, utilizing homologous materials with uniformly dispersed particles, the presented technology reduces ingredient consumption by 5–10 times due to the arrangement in chains, which enhances heat transfer efficiency in the desired direction. The present technology can be used in a variety of industrial settings, accommodating different ingredients and film thicknesses, and can be customized for various applications in electronics thermal management.

## 1. Introduction

Thermal management involves controlling heat in a system to guarantee ideal and secure operation through the utilization of conduction, convection, and radiation techniques to remove or spread out extra heat. Effective thermal regulation is crucial for a range of devices and systems like electronic devices, vehicles, power plants, imaging systems, and high-performance computing systems. Failure to properly manage heat in these devices can result in excessive heat, leading to reduced performance, a shorter lifespan, and potential damage to components. Implementing effective thermal management techniques like heat sinks, fans, liquid cooling systems, and thermal interface materials enables devices and systems to operate safely, reach peak performance, and prolong their lifespan. Deciding on the best thermal management solution depends on the particular needs of the application and achieving a balance between performance, cost, and complexity. Failure to meet these criteria may result in legal consequences, fines, or even life-threatening consequences. Poor temperature control leads to increased energy consumption, which has a negative impact on the efficiency, longevity, and environmental footprint of the device. Developing products with predefined thermal management can decrease energy consumption and lead to a more environmentally friendly device, [1,2]. Incorporating thermal control early in the design process avoids costly redesigns or repairs, [3,4]. Device reliability is guaranteed by using a variety of Thermal Interface Materials (TIMs) made of substances such as greases, fillers, adhesives, thermal conducting films, etc. They are applied through methods like potting, encapsulation, gap fillers, coatings, etc. TIMs made of metal, carbon, and polymers have the potential to achieve high thermal conductivity and show promise for cooling high-power electronics.

Thin conformal coatings in electronic devices provide initial protection for small components to avoid overheating, to be followed by degradation and oxidation. Basically, there are four main types of polymeric materials used as TIMs for the needs of electrical and electronic parts, each with unique qualities, [5]. Acrylic resins provide easy heat control and serve as a protective shield from moisture. They have high elasticity and provide good electrical insulation, convenience, and cost-efficiency, but they lack durability in tough conditions and against harsh substances. Epoxy resins (ERs) provide little thermal insulation and are mainly used to protect against moisture, abrasives (thanks to their hard surface), and chemicals. As a result, they are mainly used for potting in order to provide sturdy physical shielding. Urethane resins (URs), or polyurethane resins, provide sufficient thermal protection for a variety of applications. They are easy to use and provide excellent moisture and fuel vapor protection, along with better chemical resistance than acrylics, though they require a longer curing time. Because of their high chemical resistance, these substances may be difficult to dissolve for reworking or repairing purposes, as the necessary solvents could potentially damage the underlying components. Finally, silicone resins (SRs) are recognized for their flexibility and capability to produce tailored formulations for TIMs, whether for large objects (such as batteries) or tiny devices (such as smartphones). Their wide temperature resistance range sets them apart from the majority of urethanes, reaching up to 600 °C in certain formulations. They also offer a robust defense against chemicals, protecting against contact with outside substances. From a processing perspective, they are user-friendly (especially in highly precise manual or automated spraying), quick to cure, and rapid at ambient temperature. Hence, silicones are becoming more popular for safeguarding delicate electronics like sensors, actuators, and other components due to their exceptional wetting abilities, reducing air pockets and flaws. Silicone structural adhesives offer simple processing and deliver trustworthy, durable bonds as well as thermal shielding. Typical specifically formulated silicone elastomers are mixed with particular fillers in a composite, and common conductive fillers include metals such as silver and aluminum, carbon-based materials like carbon nanotubes and graphene, and ceramics like alumina and boron nitride (BN). Unlike metals or carbon-based materials, ceramic fillers usually possess exceptional electrical insulation properties in addition to high thermal conductivity, qualities that make them perfect for application as thermally conductive fillers in TIMs [6]. Hence, the shape and type of fillers used in thermal functional composites play a crucial role in enhancing thermal performance, indicating that the careful selection of materials is essential.

Hexagonal BN, similar to graphene, is a ceramic filler possessing a 2D structure and displaying impressive thermal conductivity and electrical insulation properties. Researchers have recently been continuously concerned with exploring the potential of BN for high-performance thermal management [7,8,9,10]. Different thermally conductive polymer composites using hexagonal boron nitride were generally examined [11,12], but also particularized by utilizing various polymeric bases such as silicone [13,14], polyurethane [15,16], polyimide [17,18], polyamide, polypropylene [19], poly(methyl methacrylate) [20], poly(ether ether ketone) [21], epoxy [22,23], polyetheretherketone [24], and poly(p-phenylene benzobisoxazole) [25]. An initial approach to improving the thermal conductivity of BN-containing materials involved combining BN with carbon nanostructures, such as graphene [26], carbon nanotubes [27], carbon nanotubes, and aluminum oxide [28], or creating hybrid structures like boron nitride-coated carbon fibers or equivalents [29,30,31,32,33]. Other hybrid structures utilizing BN were also examined, as discussed in [34,35,36]. Although such hybrid composites, as presented above, offer unique technical advantages, they also have significant technological limitations.

**Figure 1:**
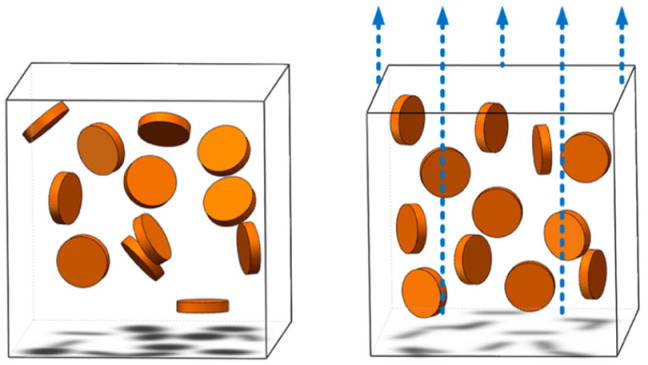
Orientation process of composites containing BN platelets.

Among all forms of presentation, BN in platelet form exhibits a higher D/t ratio, displaying significant anisotropy, with thermal conductivity strongly influenced by the orientation of the platelet. Its in-plane thermal conductivity can approximately reach 300–600 W m^−1^K^−1^, and through-plane only reaches about 30 W m^−1^ K^−1^. Therefore, the orientation of the BN platelets was anticipated to have a significant impact on the thermal conductivity of the BN-filled composites. However, research on the influence of orientation has only been thoroughly examined in recent times and continues to be a significant subject for scientists [37]. In a schematic manner, in Figure 1, the orientation process of composites containing BN platelets is presented.

Various research studies have experimented with incorporating BN particles into composites using different methods of particle orientation, such as thermal–mechanical techniques, like the orientation stacking–cutting method [38], the multi-folding and multi-laminating process [39,40], and fused deposition modeling [41]. Additionally, some studies have explored chemical–mechanical processes of particle orientation, like template-assisted chemical conversion [42] or the microfluidic spinning process with template-assisted chemical vapor deposition conversion [43]. Recently, there have been additional studies focusing on BN particle alignment through the application of magnetic fields, as seen in references [44,45]. While these procedures do reasonably enhance the thermal conductivity of BN composite structures with oriented particles, they are not easily implemented on an industrial level.

Finally, certain authors are examining how the chemical functionalization of BN particles using coupling agents can improve dispersibility, polymer chain affinity, and composite homogeneity when combined with various polymeric matrices, as discussed in references [46,47,48,49,50]. The thermal conductivity of composites typically increases by approximately 5%, and occasionally slightly more, with the addition of coupling agents. A minimum of 2% additive is necessary for a BN particle content above 10%, and structures with BN particles exceeding 25% are not advisable from technological and economic perspectives.

Based on the scientific data presented above, in the current research, the authors created BN platelet-containing silicone composites by adding carbon microfibers, incorporated at a specific percentage to improve the thermal conductivity of the composite. The composites also contained a specified percentage of silane coupling agent. The authors creatively employed DC and AC electric fields to align BN particles and carbon microfibers, based on a technique for orienting particles as described, e.g., in [51]. The new idea involved using two fields for orienting particles: a higher voltage stationary field to align the particles in chains and a lower voltage alternating field at a specific frequency to help move the particles in the polymer and facilitate the orientation, a decisive factor that enhances the uniform spread of carbon microfibers among BN in the created chains. No previous records exist of a technology using electric fields to vertically align boron nitride platelets and carbon fibers to enhance directional heat properties for heat management purposes. On the other hand, no prior research has examined the impact of simultaneously applying DC and AC electric fields to create anisotropies in composites, particularly by creating uniformly dispersed vertically aligned chains.

## 2. Materials and Methods

### 2.1. Materials

Pourable, room-temperature-cured, silicone rubber with 2 components (RTV-2) ELASTOSIL^®^ RT 620 A/B type was sourced from Wacker Chemical Corporation, Ann Arbor, MI, USA.

High-purity standard boron nitride platelets SP12 type, with a tight particle size distribution (D50 = 12 µm), were obtained from Saint-Gobain Boron Nitride, Amherst, MA, USA. In-plane thermal conductivity was 300 W m^−1^K^−1^; through-plane plane thermal conductivity was 30 W m^−1^K^−1^; and heat capacity was 0.77 J g^−1^ K^−1^ (producer datasheet).

Carbon microfibers Carbiso™ MCF type, with a fiber length < 200 µm and a diameter < 10 µm were sourced from Easy Composites Ltd., Stoke-on-Trent, UK. Thermal conductivity was 350–400 W m^−1^K^−1^ and heat capacity was 1.11 J g^−1^ K^−1^ (datasheet).

A silane-coupling agent used to form a durable bond between organic and inorganic materials, KH550 type, was sourced from Dongguan Hongrui Chemical Co. Ltd., Dongguan City, China. To simplify the technological process, the silane coupling agent was added to the composite formula while mixing the inorganic and organic materials.

Table 1 shows the details of the technological recipes, including the percentage of filler concentration indicated in weight (wt%).

### 2.2. Technological Equipment and Methods

**Figure 2:**
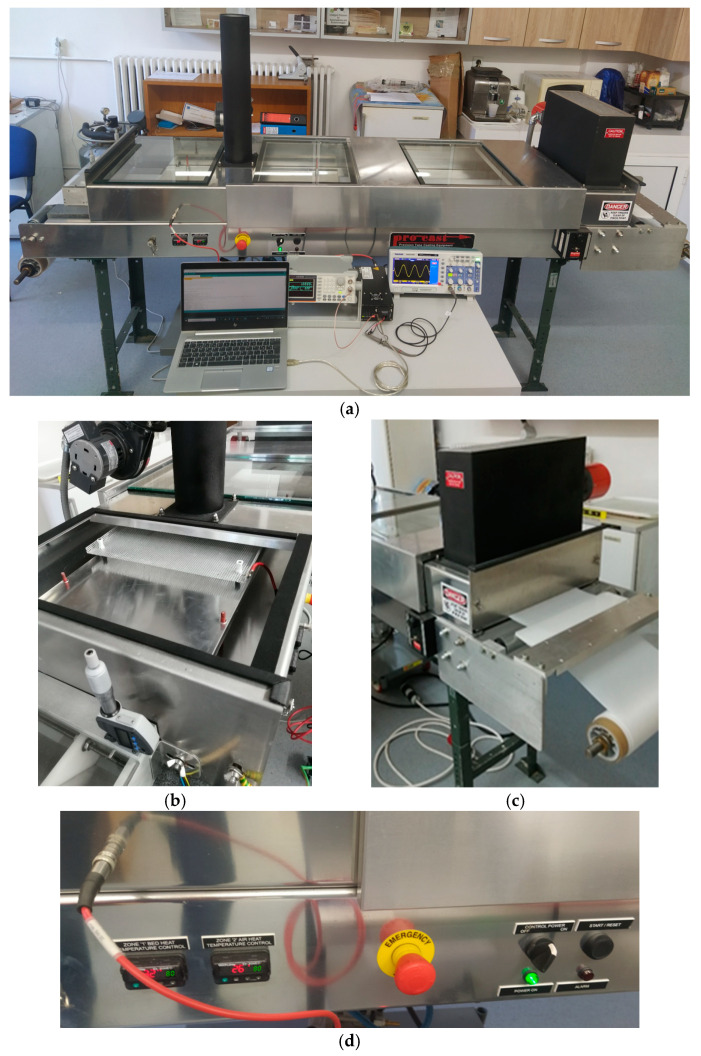
Laboratory tape caster equipment: (**a**) setup assembly; (**b**) adjustable electrodes area; (**c**) IR curing area; (**d**) curing temperature control panel.

Composite films, according to the recipes in Table 1, were produced by using a modified ATC-71LC LAB TAPE CASTING SYSTEM (HED International Inc., Ringoes, NJ, USA), as shown in Figure 2. The electrical exposure of composites was made by using a programmable signal, generated by a pulsating high-voltage source-type amplifier HA51U (Hivolt.de GmbH & Co. KG, Hamburg, Germany), and the programming was performed using the Arduino kit, an open-source microcontroller development board, [52], by use of a computer with an operating system compatible with the Arduino Uno board.

The technological process included the following main stages:

(1) Homogenization and dispersion of nano/microparticles in the liquid polymer:
  - Dosage of the components of the composite material.
  - Homogenization of the composition, carried out as ultrasonic dispersion in 5 mixing cycles with a total time of approximately 5 min.
(2) Launching of the composite film:
  - Loading the dispersion zone and adjusting the flow nozzle, depending on the desired thickness of the film.
  - Drawing the composition on a circulating PET support.
(3) Orienting the particles under an electric field:
  - Aligning the particles in the electric field, according to a programmable signal scheme; the electrical exposure was performed in our case at 1000 Vcc + 750 Vac/2.5 kHz for approximately 8 s, at an equivalent electric field intensity of approximately 18 kV/m.

The film moves between two adjustable rectangular electrodes; the space between the electrodes can range from 5 mm to 2 cm, based on the matrix and the particle content, and is also aligned with the electrical exposure to ensure the ideal intensity of the DC electric field, which firmly maintains the stability of the vertically aligned chains.

(4) Composite final curing to block particles within chains, by use of IR radiation:
  - In our case, the curing with IR radiation was performed with a 3 kW heater, for approximately 4 s; the curing temperature is automatically controlled and adjusted.
  - Unwinding the film and pulling it onto the reel; the film speed is automatically controlled, and it was approximately 0.05 m/s in the presented case, to guarantee the efficient alignment and curing of particles within the previously stated time frames.

Because carbon microfibers are up to 200 µm in length, the research was conducted on composites that were 1 mm thick to ensure that the fibers were adequately dispersed and integrated. The final samples were cut from a film that was 40 cm wide, depending on the analysis needed for each sample.

It is important to point out that the technological settings mentioned above align with the recipes listed in Table 1 for a film with a maximum thickness of 1 mm.

In terms of technology, the voltage values and frequency are determined by the type and concentration of fillers. These parameters need to be determined through experimentation before successfully launching a material with properly aligned chains of particles. Also, the duration of exposure to electric fields and IR radiation necessary for curing is significantly influenced by the thickness of the film. According to the technical capabilities of the equipment, the thickness of the film can be adjusted to a greater extent, e.g., from 50 µm to 2 mm, sizes commonly utilized for the thermal management of electronic devices [6], and covering various thermal management applications in the electronics field.

### 2.3. Testing Equipment and Methods

Optical scanning microscopy SEM and energy-dispersive X-ray spectroscopy (EDX) were performed with a field emission and focused ion beam scanning electron microscope (SEM) model Tescan Lyra III XMU (Libušina tř. 21 623 00, Brno-Kohoutovice, Czech Republic). Structural characterization was carried out by X-ray diffraction (XRD) using CuKα radiation with the Ni filter Bruker AXS D8 Advance with CuKα radiation (λ = 0.154 nm). Diffraction patterns were recorded at room temperature in Bragg–Brentano geometry at an angle 2θ of 20° to 65° at a rate of 0.6°/min (2θ)/min.

Laser Flash Analyzer equipment, model LFA457, from NETZSCH-Gerätebau GmbH, Selb, Germany, was used to determine the thermal conductivity; 5 determinations were made for each sample at the same temperature of 25 °C.

The dielectric features were determined by broadband dielectric spectroscopy with a Solartron 1260 A dielectric spectrometer (Solartron Analytical, Farnborough, UK). The measurements were conducted with an AC voltage amplitude of 3 V over a frequency range of 1–10 kHz, using a measuring electrode with a diameter of 30 mm.

Tensile tests were carried out at room temperature (22 °C), using a universal testing machine (Instron, Norwood, MA, USA), at a speed of 30 mm/min. Three replicates were used for each sample to obtain the averaged values and standard deviation. Tensile strength values were directly determined from the stress–strain curves.

## 3. Results and Discussion

### 3.1. SEM Analysis

**Figure 3:**
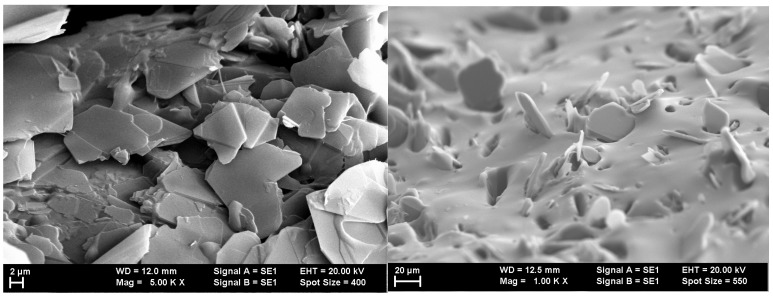
BN platelets before and after integration into composites with uniformly distributed particles.

**Figure 4:**
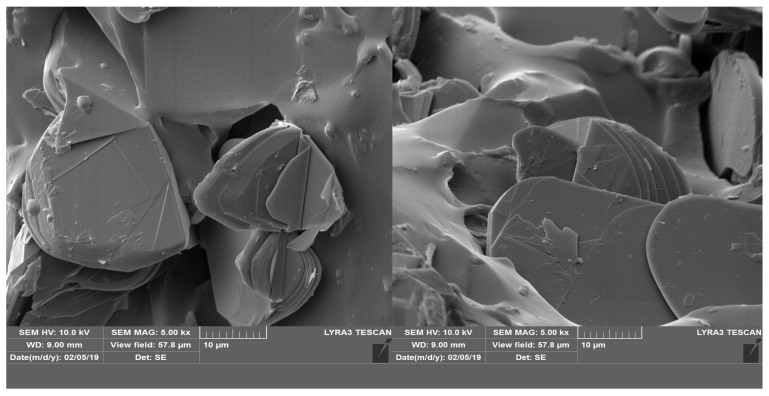
Internal structure of composite R1, with 12% BN platelet (uniformly distributed particles).

**Figure 5:**
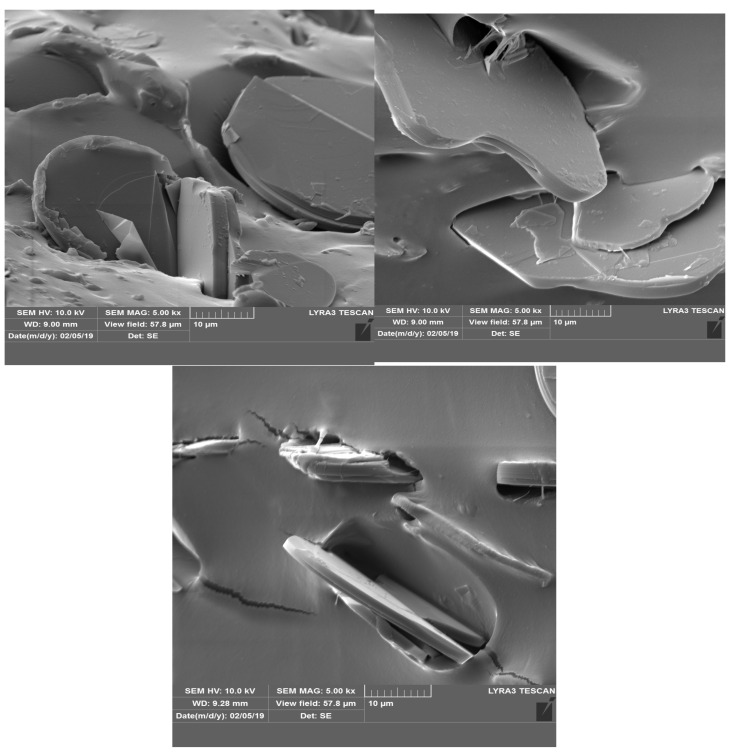
Internal structure of composite R2, with 12% BN platelet (oriented particles, aligned along a chain).

The analysis using SEM is important for evaluating the composition structure and effectiveness of the orientation method with the tailored electric field. Figure 3 shows the composition and size of BN platelets before being processed for compounding, as well as their integration into composites, specifically for the R1 sample. Figure 4 and Figure 5 present the comparison between uniform dispersion and chain formation, specifically for R1 and R2 samples. For R2, the BN particles are seen moving closer together and forming a vertical chain in a gradual process. The key point is that the orientation in chains occurs along the length of the platelet, potentially enhancing thermal conductivity.

**Figure 6:**
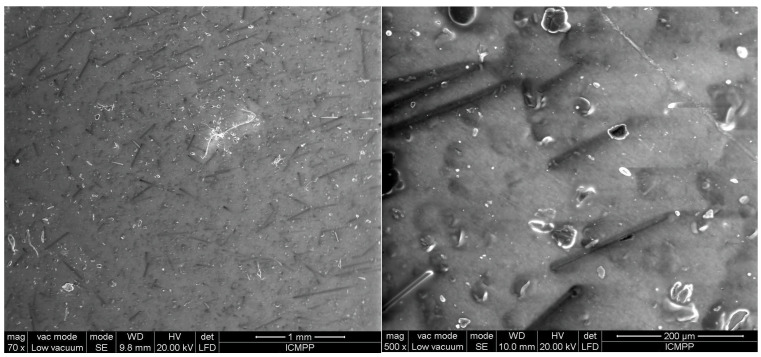
SEM images for sample R3.

**Figure 7:**
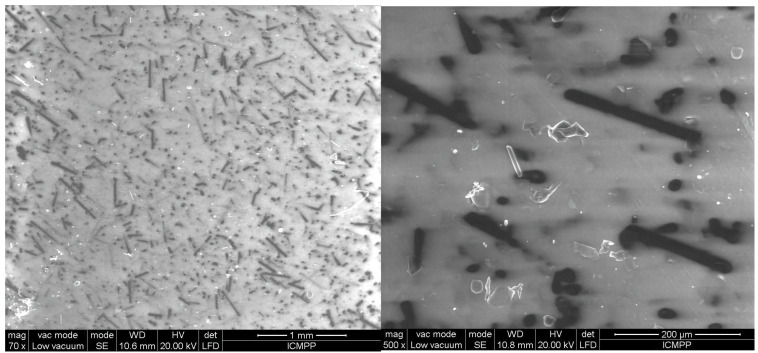
SEM images for sample R4.

**Figure 8:**
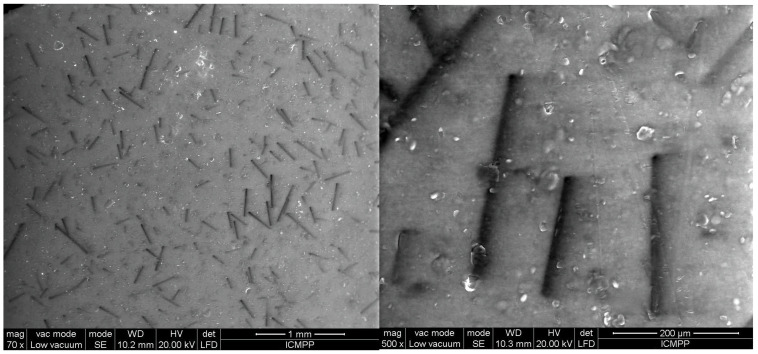
SEM images for sample R5.

**Figure 9:**
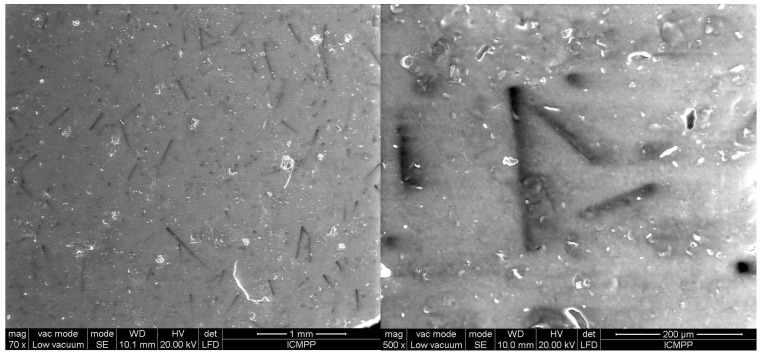
SEM images for sample R6.

**Figure 10:**
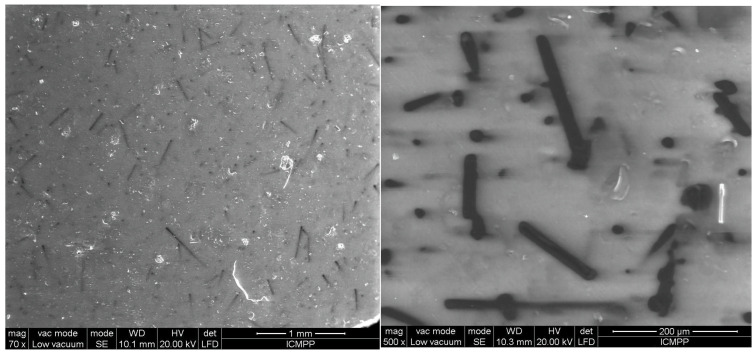
SEM images for sample R9.

Figure 6, Figure 7, Figure 8, Figure 9 and Figure 10 display the arrangement of composites containing varying levels of carbon microfibers, at magnifications of 70 and 500, which are deemed the most straightforward for visualizing the distribution of particles in composites. The significance of the analysis is in showcasing how the chains are evenly spread across the sample’s surface, fully developed, and mature.

By comparing Figure 6 and Figure 7, it is evident that there is a formation of chains with a higher particle concentration on the material surface in Figure 7 and fewer horizontal carbon microfibers. The higher level of carbon microfibers (2%) and lower BN content (10%) make the carbon microfiber orientation more challenging in this scenario, with some carbon microfibers not being attached to the chains and remaining horizontal.

The outcomes appear alike for both R5 and R6, as shown in Figure 8 and Figure 9 compared to Figure 6 and Figure 7, but here, the carbon microfibers’ orientation is more facile due to their lower content (1%) at a comparable BN content (here 12.5%). However, it can be noted that there are still areas where the carbon microfibers are not completely absorbed in the chains, with some remaining partially attached horizontally to the surface of the material. When Figure 7 and Figure 9 are compared at 70 magnification, it is clear that there are fewer chains in the composite with fewer carbon microfibers.

**Figure 11:**
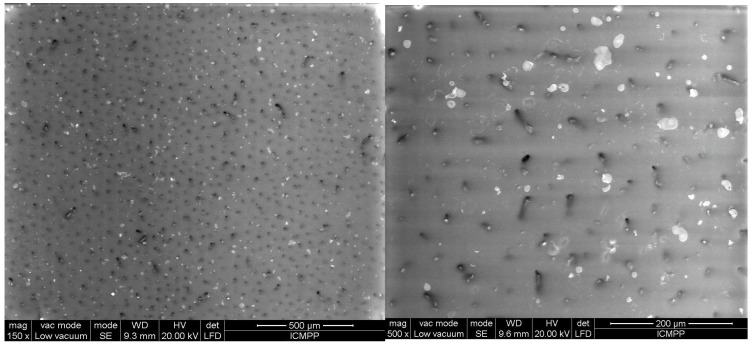
SEM images for sample R10.

Figure 10 and Figure 11 represent samples R9 and R10, containing a greater number of BN platelets (15%) and a lower number of carbon microfibers (0.5%). Through the process of chain formation, we observed that nearly all carbon microfibers were trapped in chains, primarily aligned vertically. In this scenario, the number of chains is higher because of the higher amount of BN, which more easily integrates with the carbon microfibers. Considering the aforementioned findings, it could be observed that the partial incorporation of carbon microfibers into chains is influenced by two factors: the number of fibers, which should be put in relation to the material thickness due to the relatively high fiber length, and the concentration of BN platelets, which aid in the absorption of fibers into chains and facilitate their alignment along the chain. Therefore, assuming a material thickness of 1 mm, the most suitable composition is considered to be a minimum of 15% BN platelets and approximately 0.5% carbon microfibers to achieve a reasonable uniformity in terms of their chain formation and distribution on the material surface.

**Figure 12:**
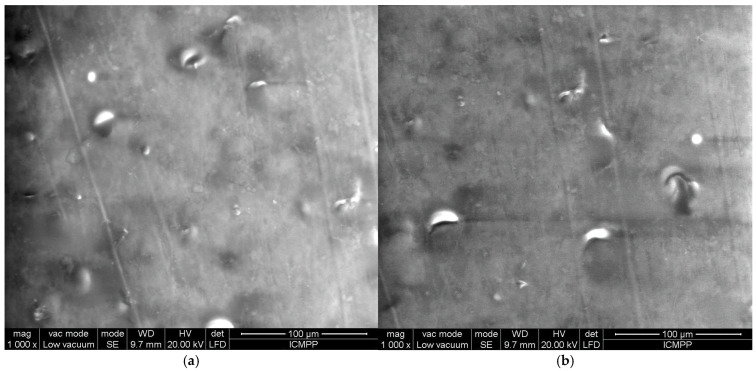
Comparative SEM images for samples: (**a**) R11 and (**b**) R12.

Seeing the chain formation in composites with BN platelets alone is also beneficial, as shown in Figure 12 for R11 and R12, which have the highest BN content (20%). The even distribution of particles is clearly seen in Figure 12a, while Figure 12b shows the formation of chains that are more uniform and denser than those in composites with carbon microfibers. In general, for all recipes containing oriented particles, it should be noted that the chains are evenly scattered across the sample surface and are fully formed and developed.

### 3.2. X-Ray Diffraction Analysis

**Figure 13:**
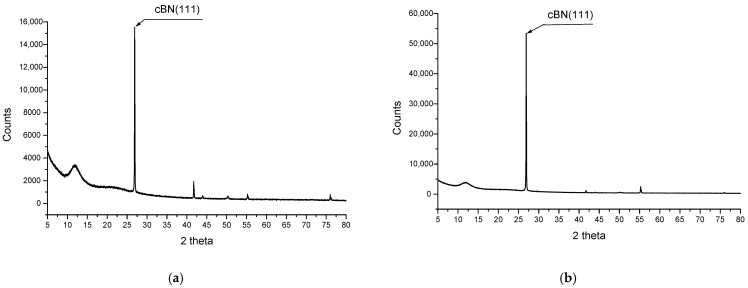
Comparative XRD analysis for samples (**a**) R1 and (**b**) R2.

**Figure 14:**
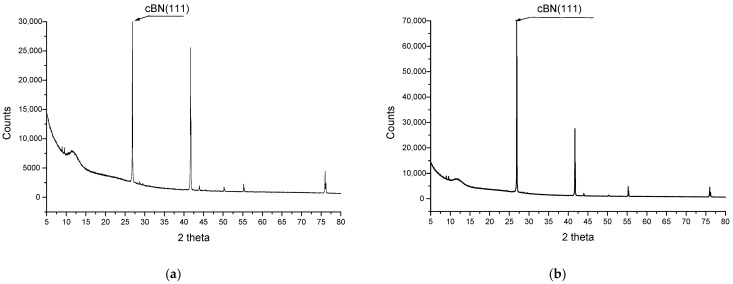
Comparative XRD analysis for samples (**a**) R5 and (**b**) R6.

X-ray diffraction analysis certifies the chains’ formation by aligning the BN platelets and carbon microfibers in the electrical field direction. The results are presented in Figure 13 for R1 and R2 (containing BN platelets only) and Figure 14 for R5 and R6 (containing BN platelets and carbon microfibers).

It is obvious that for the composites with oriented particles, the intensity of the diffraction maximum (111) of BN is approximately 3.4 times greater than for composites with a uniformly distributed orientation in the case of R2 compared to R1 (Figure 13) and about 2.3 times higher in the case of R6 compared R5 (Figure 14). The texture of the structure is clearly pronounced along the (111) axis, validating the structure seen through scanning electron microscopy. The other picks (for silicon, carbon, etc.) remain unchanged under the alignment process. Regarding BN, the intensity of the diffraction differs from samples R1 to R5 due to a higher content of BN platelets in the case of R5. However, the reduction in diffraction enhancement from R2 (Figure 1) to R6 (Figure 14) is related to the chain architecture modification, because, e.g., in the case of R6, the presence of carbon microfibers partially alters the alignment of BN platelets.

### 3.3. EDX Analysis

**Figure 15:**
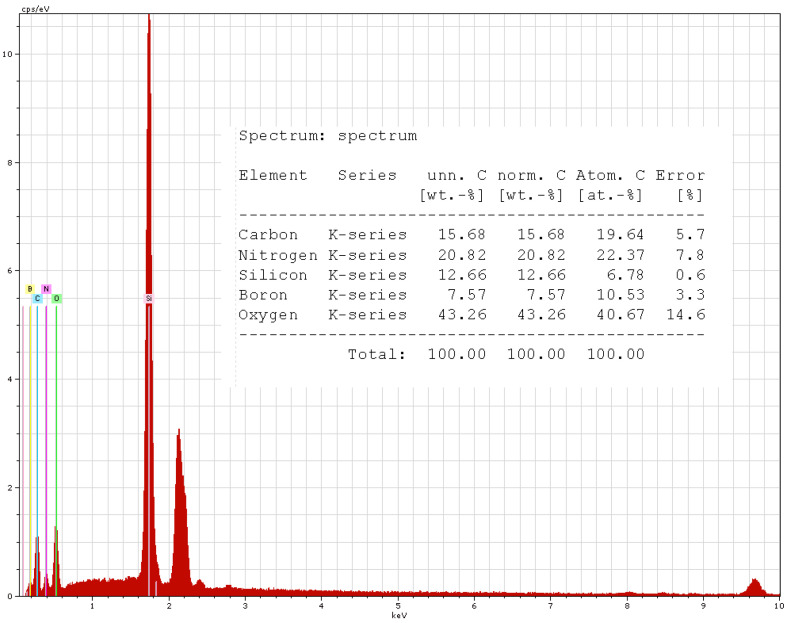
EDX results for R3.

**Figure 16:**
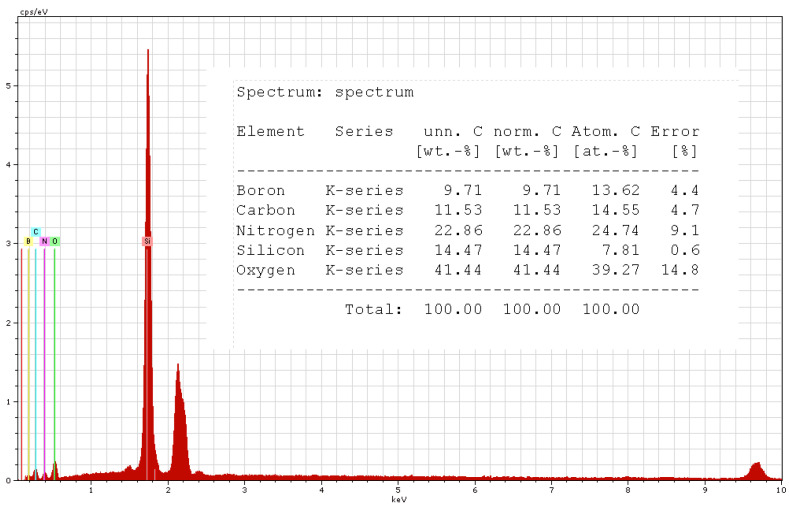
EDX results for R5.

The compositions of the samples were investigated using a scanning electron microscope. Only two samples were included in the results because their composition was very similar, i.e., for R3 (with 10% BN platelet addition and 2% carbon microfiber addition) in Figure 15 and for R5 (with 12.5% BN platelet addition and 1% carbon microfiber addition) in Figure 16. The comparative variations in C and B are emphasized. However, a key finding was that no residual technological impurities such as Al, S, etc., were discovered in the analysis. This outcome enables a more accurate assessment of the analysis of the dielectric parameters that will be further addressed.

### 3.4. Physical–Mechanical Features

Table 2 presents the various physical and mechanical properties of composites. The density stays the same for both uniform and oriented samples with the same composition. A higher density is linked to a higher number of BN platelets, particularly R11–12, and decreases when there is a lower BN platelet content and more carbon microfibers, eventually reaching its lowest value in samples R3–R4.

The Shore hardness is unexpectedly lower in composites with oriented particles, regardless of their composition, possibly because of the prevalent silicone structure in the sample surface. When the dispersion is uniform, the material with the most BN platelets (specifically R11–12) also has the highest hardness. However, when it comes to materials containing oriented particles, it appears that the number of carbon microfibers has a significant impact; for instance, samples like R4, R6, and R10 show higher values in comparison to R12. Another intriguing finding is that the hardness of samples containing solely BN-oriented particles remains consistently similar, regardless of the particle concentration.

The impact of the composition upon tensile strength values is comparable to that upon Shore hardness, with the values being generally reduced in composites with oriented particles, regardless of their composition. The number of carbon microfibers greatly affects the tensile strength of composites with either uniform or particle orientations. The samples containing more carbon microfibers (R3–R4, with 2% content) exhibit the highest tensile strength, followed by R5–R6 (with 1% carbon microfibers’ content), which have slightly lower strength values. These tensile strength findings align with the data shown in [50].

### 3.5. Dielectric Characterization

**Figure 17:**
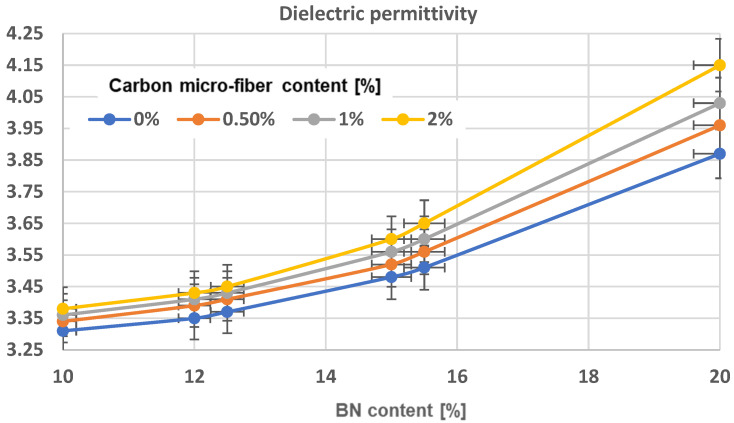
Dielectric permittivity characteristics of composites with uniform dispersion.

**Figure 18:**
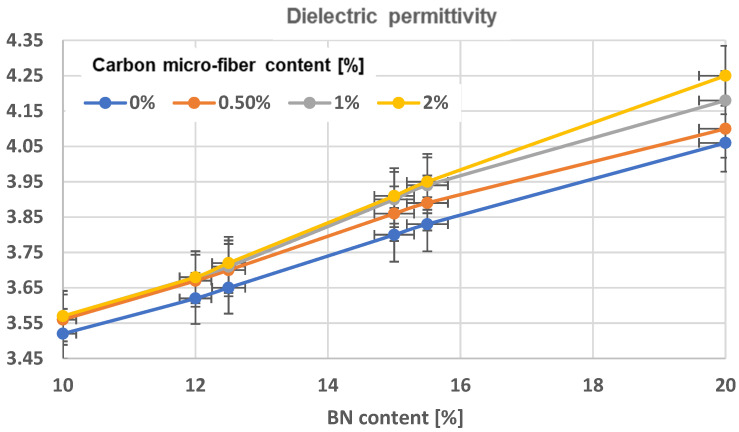
Dielectric permittivity characteristics of composites with oriented particles.

The permittivity and loss factor (TgDelta) dielectric parameters were calculated based on IEC60250 [53] standards, using a reference frequency of 1 kHz. The data are shown in Table 3. An obvious increase in permittivity values is observed in all composites with aligned particles, regardless of their composition, Figure 17, comparing to Figure 18. An increase in the number of BN platelets leads to a higher level of dielectric permittivity enhancement, though this enhancement is saturated when concentrations surpass 15%. The reason for this is that the dielectric permittivity of bulk BN platelets varies from 3.29 to 3.76 in the out-of-plane direction (with thickness dependency) and remains relatively constant at 6.82 to 6.93 in-plane. Thus, aligning them in the electric field promotes an in-plane orientation within chains, leading to a noticeable increase in permittivity. However, not all platelets in the chain are oriented in-plane. As the concentration of platelets increases, achieving in-plane orientation becomes more difficult. Therefore, at a 15% concentration of BN platelets, we can achieve an optimal orientation. Analyzing the table alone makes it difficult to fully explain the impact of carbon microfibers but comparing samples like R2 and R6 does show a positive effect. Alternatively, the inclusion of carbon microfibers could also positively impact uniform composites when comparing R1 to R3, R5, or R9. To understand how the material’s composition affects permittivity, an extended analysis was performed, as shown in Figure 12 (for composites with uniformly dispersed particles) and Figure 13 (for composites with oriented particles). The impact of incorporating carbon microfibers on the dielectric permittivity of homogeneous composites is clearly beneficial, especially when the BN content surpasses 12%. For instance, the permittivity value of a material containing 15.5% BN and 2% carbon microfibers is comparable to that of a material with 20% BN and without fibers. The technological advantage of producing materials with microfibers could be ongoing as the price of BN platelets is at least 10 times greater than carbon microfibers. For composites with oriented particles, the impact of carbon microfiber addition is significant at all concentrations of BN. However, at higher fiber content, the effect becomes more beneficial at higher BN concentrations, which correlates well with the previous SEM observations in Figure 6, Figure 7, Figure 8, Figure 9, Figure 10, Figure 11 and Figure 12, related to the fact that the integration of carbon microfibers within chains depends on the BN concentration. The impact of incorporating carbon microfibers on the dielectric permittivity is significant, especially when the BN content surpasses 15%. Nonetheless, it can be inferred that a material consisting of 12% BN and 2% carbon microfibers exhibits comparable permittivity to one with 15% BN, or a material comprising 15% BN and 2% carbon microfibers has equivalent permittivity lower than a material with 18% BN, resulting in similar economic implications for materials technology in terms of reducing the costly BN platelet content.

The examination of the dielectric loss factor (TgDelta) indicates a slight distinction between uniformly and particle-oriented composites across all formulations. The findings in reference [54] that aligning the BN platelets can lower dielectric loss by inhibiting defect ions perpendicular to the field direction were not verified; however, this may only hold true at elevated temperatures, which does not apply to our materials. Samples with carbon microfibers show significantly higher dielectric loss compared to samples with only BN, such as reaching 0.0102 for the sample containing 2% microfiber content. Ultimately, the resistivity values were examined. For samples containing only BN, the resistivity decreases slightly as the BN content increases, and there is minimal difference between similar recipes with either uniformly or oriented particles, as also noticed in [55]. Noticeable variations are clearly observed in the samples that have microfibers. In this case, the resistivity is at least 10^4^ times less compared to samples containing only BN. Conversely, the alignment of particles once more decreases the resistance by at least 10^3^ times. The sample with a 2% concentration of microfibers reached the minimum value of 4.15 × 10^5^ Ohm m. The reason is that with less BN, microfibers align vertically through the BN platelets, creating parallel microconductive areas within the chains and reducing the material’s electrical resistance overall.

### 3.6. Thermal Conductivity Analysis

**Figure 19:**
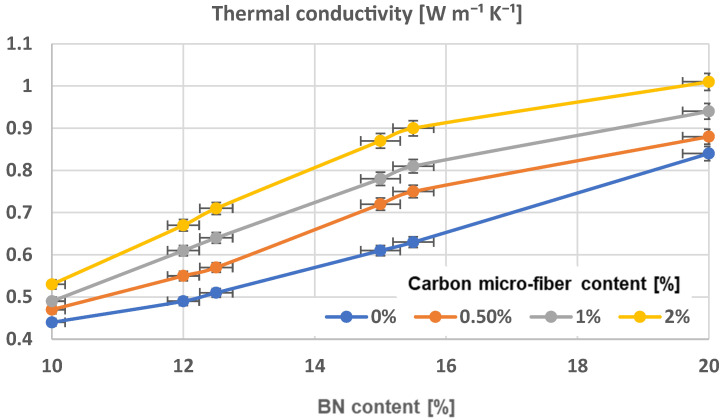
Thermal conductivity characteristics of composites with uniform dispersion.

**Figure 20:**
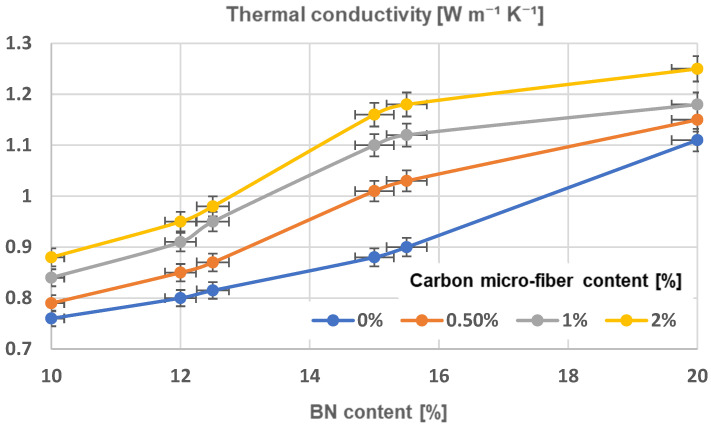
Thermal conductivity characteristics of composites with oriented particles.

The measurement of thermal conductivity using the Laser Flash Method and its corresponding time–temperature curve is viewed as more effective for analyzing samples with a greater surface area and thickness within the range of the materials presented, in comparison to alternative methods, e.g., the 3ω method [56]. Table 4 displays the thermal conductivity values of composite samples. The difference in thermal conductivity of composites is linked to the architecture of chains when the thermal conductivity of carbon microfibers is similar to boron nitride platelets, as previously discussed in terms of resistivity characteristics. Thermal conductivity was significantly improved in all samples containing aligned particles, regardless of their composition, Figure 19, comparing to Figure 20. Overall, the obtained data align with the findings in, for instance, the study referenced in [42]. The sample with 20% BN platelets content had the highest thermal conductivity value of 1.11 W m^−^^1^ K^−^^1^, while the sample with 2% carbon microfiber content showed the greatest thermal conductivity enhancement. Typically, the thermal conductivity enhancement decreases for composites with higher particle content, despite the fact that thermal conductivity generally increases with higher particle content in absolute terms, meaning that the alignment process remains more relevant at a lower content of particles. The addition of carbon microfibers in composites has a positive impact on enhancing thermal conductivity. In order to analyze how the composition influences the thermal conductivity, an extended analysis was conducted and the findings are displayed in Figure 14 (for uniform composites) and Figure 15 (for composites with aligned particles). For uniform composites, adding carbon microfibers greatly improves thermal conductivity, especially with a BN content of around 15%. The rise continues at a higher BN content but the effect is progressively limited. When oriented particles are present in composites, adding carbon microfibers significantly boosts thermal conductivity, with noticeable enhancements even at a BN content above 12%. The maximum is reached at 15% BN content. However, once the BN content exceeds 15.5%, the impact is progressively limited too. The reason can be found in the architecture of the materials that have microfibers. In instances of even distribution, the microfibers, which are much longer than BN platelets, spread out randomly throughout the material, forming various orientations that help create quicker heat pathways. In this scenario, higher concentrations of microfibers result in increased thermal dissipation. In the case of composites with oriented particles, adding carbon microfibers to chains typically enhances heat transfer pathways by clustering within the chains. With a reduced amount of BN, the microfibers orient vertically between the BN platelets, forming parallel microconductive regions for heat transfer, and additional microfibers enhance this effect. However, when the BN content is increased, a saturation process happens as the concentration of fibers increases because the fibers within chains are only partially oriented vertically, as BN platelets take priority in vertical orientation. These assumptions align perfectly with the observations provided alongside the SEM images in Figure 6, Figure 7, Figure 8, Figure 9, Figure 10, Figure 11 and Figure 12.

The findings in this study showed slightly inferior results for composites with oriented BN platelets and carbon microfibers when compared to previous results presented in the literature, such as those in [57], which described a similar composition and may be the most relevant to our paper but explores a totally different and more complicated chemical–mechanical technology for particle alignment. Similar structures involving BN particles but other types of carbon structures were also thoroughly examined, as noted in references [32,58,59,60,61,62,63,64], although comparing their findings to ours is more indicative due to the fact that the proportion of carbon structures is over 15 wt% in those instances. When compared to [58], research that used 10 wt% spherical BN particles and a much higher content of 10–30 wt% carbon microfibers, with a uniform dispersion of additives. In that research, the composite presented a value of thermal conductivity of 1.03 W m^−^^1^ K^−^^1^, similar to the value achieved within our technology at 15 wt% BN and only 0.5 wt% carbon microfibers. It is technically crucial to limit the number of carbon particles or fibers as they can significantly raise the material’s dielectric loss and conductivity, thus restricting their use in electronic applications requiring high electrical insulation performance. On the other hand, structures with BN particles exceeding 25% are not advisable for technological and economic reasons. From this perspective, the composites presented could cover a wider range of applications in the thermal management of electronics, mainly due to the continuous miniaturizing of electronic equipment. Compared to actual technologies using homolog materials but with a uniform dispersion of particles, with our technology, ingredient consumption can be reduced by 5–10 times, providing numerous advantages, especially when working with costly powders of certain sizes as they are now organized in chains, ensuring efficient heat transfer in the intended orientation. Ultimately, only the properties of the chain matter for achieving superior electro-thermal properties, compared to materials with evenly dispersed particles.

The main benefit of the study is that the presented technology can be easily expanded for industrial use, it is flexible in terms of ingredients and film thickness, and it can be customized for various thermal management applications in the electronics field. Electronic Thermal Management Materials Market size achieved USD 6.4 billion in 2023 and will grow at 7.5% CAGR from 2024 to 2032 [65], offering clear opportunities. The targeted application of the presented technology is related to, e.g., thermally radiative materials for microelectronics, elastomeric pads for electric autonomous vehicles, interface materials for miniaturized and/or portable healthcare devices, adhesive tapes for thermal interface in telecommunication devices, etc. [66].

When we compare our findings with homolog results from previous studies, such as [45,67,68,69], the performance demonstrated by the materials we presented is comparable, exhibiting a thermal conductivity of approximately 1 W m^−1^ K^−1^, yet with a significantly lower content of BN particles. However, the respective technologies, even when employing magnetic fields for the orientation of BN particles, have the limitation of low productivity, questionable reproducibility in larger-scale manufacturing, and no viable equipment to efficiently produce such materials for the market. In our scenario, we showcased a pilot-scale apparatus that can generate substantial numbers of consistent composite forms featuring aligned particles, thus proving its industrial feasibility through a roll-to-roll process. Concerning the environmental impact, the compact nature of our equipment, associated with minimal energy consumption for alignment processes and curing, provides evident advantages for the carbon footprint.

## 4. Conclusions

This study presents silicone composites with uniform dispersion and electrically oriented boron nitride platelets and carbon microfibers. The technological process included the homogenization and dispersion of nano/microparticles in the liquid polymer, orienting the particles under an electric field, and composite final curing to block particles within chains via the use of IR radiation.

The innovative idea involved using two fields for orienting particles: a higher-voltage stationary field to align the particles in chains and a lower-voltage alternating field at a specific frequency to help move the particles in the polymer and facilitate the orientation, a decisive factor that enhances the uniform spread of carbon microfibers among BN in the created chains.

According to SEM images, it should be noted that the chains are evenly scattered across the sample surface and are fully formed and developed, but their architecture critically depends on the composition. There are fewer chains in the composite with fewer carbon microfibers. The texture of the structure was confirmed through X-ray diffraction analysis, which revealed alignment along the BN(111) axis, supporting the structure observed with scanning electron microscopy.

A higher density is linked to a higher number of BN platelets, which decreases when there is a lower BN platelet content and more carbon microfibers. The Shore hardness is unexpectedly lower in composites with oriented particles, regardless of their composition, possibly because of the prevalent silicone structure in the sample surface. When the dispersion is uniform, the material with the most BN platelets has the highest hardness. The tensile strength values are generally reduced in composites with oriented particles, regardless of their composition. The number of carbon microfibers greatly affects the tensile strength of composites with either a uniform or particle orientation. The samples containing more carbon microfibers exhibit the highest tensile strength.

An obvious increase in permittivity values is observed in all composites with aligned particles, regardless of their composition. The higher the concentration of BN platelets, the greater the enhancement, but the effect decreases gradually after reaching a concentration of 15%. The impact of incorporating carbon microfibers on the dielectric permittivity of homogeneous composites is clearly beneficial, especially when the BN content surpasses 12%. The value of a material containing 15.5% BN and 2% carbon microfibers is comparable to that of a material with 20% BN and without fibers. The technological advantage of producing materials with microfibers could be ongoing as the price of BN platelets is at least 10 times greater than carbon microfibers. The examination of the dielectric loss factor (TgDelta) indicates a slight distinction between uniform and oriented-particle composites across all formulations. For samples containing only BN, the resistivity decreases slightly as the BN content increases, and there is minimal difference between similar recipes with either uniformly or oriented particles. Noticeable variations are clearly observed in the samples that have microfibers, with the resistivity being at least 10^4^ times lower compared to samples containing only BN. Conversely, the alignment of particles once more decreases the resistance by at least 10^3^ times.

The difference in thermal conductivity of composites is linked to the architecture of chains. Thermal conductivity was significantly improved in all samples containing aligned particles, regardless of their composition. For uniform composites, adding carbon microfibers greatly improves thermal conductivity, especially with a BN content of over 12%. The greatest rise occurred with the addition of carbon microfibers at 2% when the BN content exceeds 15.5%. When oriented particles are present in composites, adding carbon microfibers significantly boosts the thermal conductivity, with noticeable enhancements even at a BN content above 12%. However, once the BN content exceeds 15.5%, the impact becomes less relevant because the fibers within chains are only partially oriented vertically, as the BN platelets take priority in a vertical orientation.

The results of this research demonstrated better outcomes for composites containing BN platelets and carbon microfibers than previous results presented in the literature, while also using a simpler technology for processing the polymer matrix and aligning particles.

Limiting the number of carbon particles or fibers is essential to prevent a notable increase in the material’s dielectric loss and conductivity, which hinders their suitability for electronic applications that demand strong electrical insulation. However, it is not recommended to use structures containing more than 25% BN particles for technological and economic purposes.

In contrast to current technologies, by utilizing homologous materials with uniformly dispersed particles, our technology reduces ingredient consumption by 5–10 times. This offers several benefits, particularly when using expensive powders of specific sizes that are now arranged in chains to enhance heat transfer efficiency in the desired direction. We demonstrated a pilot-scale device capable of producing significant quantities of uniform composite structures with aligned particles, thereby confirming its industrial viability via a roll-to-roll method. Regarding the ecological effect, the compact design of our devices, coupled with the low energy usage for alignment and curing, offers clear benefits for the carbon footprint. The presented technology is highly versatile for industrial applications, able to accommodate different ingredients and film thicknesses, and can be tailored for various uses in electronics thermal management, e.g., thermally radiative materials for microelectronics, elastomeric pads for electric autonomous vehicles, interface materials for miniaturized and/or portable healthcare devices, adhesive tapes for thermal interface in telecommunication devices, etc.

## Figures and Tables

**Table 1:** Recipe description.

No.	Recipe	Structure
R1	12% BN platelet; 2% KH550	Uniform
R2		Oriented
R3	10% BN platelet; 2% carbon microfibers; 2% KH550	Uniform
R4		Oriented
R5	12.5% BN platelet; 1% carbon microfibers; 2% KH550	Uniform
R6		Oriented
R7	15.5% BN platelet; 2.5% KH550	Uniform
R8		Oriented
R9	15% BN platelet; 0.5% carbon microfibers; 2.5% KH550	Uniform
R10		Oriented
R11	20% BN platelet; 2.5% KH550	Uniform
R12		Oriented

**Table 2:** Mechanical features of composites.

No.	Density [g cm^−3^]	Shore Hardness	Tensile Strength [MPa]
R1	1.27	36	2.86
R2		34	2.14
R3	1.26	38	3.85
R4		36	3.23
R5	1.28	40	3.54
R6		37	2.87
R7	1.31	39	2.74
R8		34	2.05
R9	1.30	38	3.32
R10		36	2.59
R11	1.33	42	2.63
R12		34	1.84

**Table 3:** Dielectric parameters of composites.

No.	Recipe	Permittivity	TgDelta	Resistivity [Ohm m]	Permittivity Enhancement
R1	12% BN platelet; 2% KH550	3.35	0.0077	4.86 × 10^13^	8%
R2		3.62	0.0078	8.21 × 10^12^	
R3	10% BN platelet; 2% carbon microfibers; 2% KH550	3.37	0.0098	2.33 × 10^8^	6.2%
R4		3.58	0.0102	4.15 × 10^5^	
R5	12.5% BN platelet; 1% carbon microfibers; 2% KH550	3.43	0.0086	4.44 × 10^9^	8.2%
R6		3.71	0.0088	7.22 × 10^6^	
R7	15.5% BN platelet; 2.5% KH550	3.51	0.0076	3.18 × 10^13^	9.7%
R8		3.85	0.0077	6.47 × 10^12^	
R9	15% BN platelet; 0,5% carbon microfibers; 2.5% KH550	3.54	0.0082	5.27 × 10^10^	9.3%
R10		3.87	0.0083	9.53 × 10^7^	
R11	20% BN platelet; 2.5% KH550	3.72	0.0075	2.76 × 10^13^	9.2%
R12		4.06	0.0076	4.82 × 10^12^	

**Table 4:** Thermal parameters of composites.

No.	Thermal Conductivity K [W m^−^^1^ K^−^^1^]	Thermal Conductivity Enhancement
R1	0.49	63.2%
R2	0.80	
R3	0.53	66%
R4	0.88	
R5	0.64	48.4%
R6	0.95	
R7	0.63	42.9%
R8	0.90	
R9	0.72	40.3%
R10	1.01	
R11	0.84	32.1%
R12	1.11	

## Data Availability

The original contributions presented in the study are included in the article, further inquiries can be directed to the corresponding author.
